# Surgical management of Encapsulating Peritoneal Sclerosis (EPS) in children: international case series and literature review

**DOI:** 10.1007/s00467-021-05243-0

**Published:** 2021-08-26

**Authors:** Videha Sharma, Zia Moinuddin, Angela Summers, Mohan Shenoy, Nicholas Plant, Semir Vranic, Agnieszka Prytula, Zlatan Zvizdic, Vasiliki Karava, Nikoleta Printza, John Vlot, David van Dellen, Titus Augustine

**Affiliations:** 1 Department of Renal and Pancreas Transplantation, Manchester Royal Infirmary, Manchester University NHS Foundation Trust, Oxford Road, Manchester, M13 9WL UK; 2 Royal Manchester Children’s Hospital, Manchester University NHS Foundation Trust, Manchester, UK; 3 College of Medicine, QU Health, Qatar University, Doha, Qatar; 4 Biomedical and Pharmaceutical Research Unit, QU Health, Qatar University, Doha, Qatar; 5 Paediatric Nephrology and Rheumatology Department, Ghent University Hospital, Ghent, Belgium; 6 Clinical Centre University of Sarajevo, Sarajevo, Bosnia and Herzegovina; 7 Aristotle University of Thessaloniki, Thessaloniki, Greece; 8 Sophia Children’s Hospital, Erasmus MC: University Medical Centre, Rotterdam, The Netherlands; 9 Division of Diabetes, Endocrinology and Gastroenterology, University of Manchester Faculty of Biology, Medicine and Health, Manchester Academic Health Science Centre, Manchester, UK

**Keywords:** Kidney failure, Peritoneal dialysis, Encapsulating Peritoneal Sclerosis, Surgery

## Abstract

**Background:**

Encapsulating Peritoneal Sclerosis (EPS) is a rare phenomenon in paediatric patients with kidney failure treated with peritoneal dialysis (PD). This study highlights clinical challenges in the management of EPS, with particular emphasis on peri-operative considerations and surgical technique.

**Methods:**

Retrospective analysis of all paediatric patients with EPS treated at the Manchester Centre for Transplantation.

**Results:**

Four patients were included with a median duration of 78 months on PD. All patients had recurrent peritonitis (> 3 episodes), and all had symptoms within three months of a change of dialysis modality from PD to haemodialysis or transplant. In Manchester, care was delivered by a multi-disciplinary team, including surgeons delivering the adult EPS surgical service with a particular focus on nutritional optimisation, sepsis control, and wound management. The surgery involved laparotomy, lavage, and enterolysis of the small bowel + / − stoma formation, depending on intra-abdominal contamination. Two patients had a formal stoma, which were reversed at three and six months, respectively. Two patients underwent primary closure of the abdomen, whereas two patients had re-look procedures at 48 h with secondary closure. One patient had a post-operative wound infection, which was managed medically. One patient’s stoma became detached, leading to an intra-abdominal collection requiring re-laparotomy. The median length of stay was 25 days, and patients were discharged once enteral feeding was established. All patients remained free of recurrence with normal gut function and currently two out of four have functioning transplants.

**Conclusions:**

This series demonstrates 100% survival and parenteral feed independence following EPS surgery. Post-operative morbidity was common; however, with individualised experience-based decision-making and relevant additional interventions, patients made full recoveries. Health and development post-surgery continued, allowing the potential for transplantation.

**Graphical abstract:**

A higher resolution version of the Graphical abstract is available as Supplementary information 
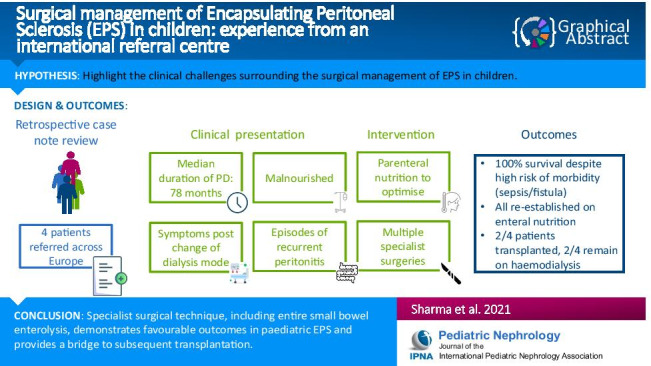

**Supplementary Information:**

The online version contains supplementary material available at 10.1007/s00467-021-05243-0.

## Introduction

Encapsulating Peritoneal Sclerosis (EPS) is a rare but potentially devastating diagnosis in paediatric patients with kidney failure treated with peritoneal dialysis (PD) [1]. The prevalence of EPS in adult PD registries ranges from 0.5 to 2.5% [2–4]. Characteristic features of EPS include chronic inflammation and thickening of the peritoneum with encapsulation of bowel loops and formation of a pathognomonic fibrotic cocoon [5]. It has a spectrum of symptoms, ranging from general malaise, weight loss, and anorexia to eventual bowel obstruction, manifesting with abdominal pain, vomiting, and constipation [6]. Over the course of the disease, patients may lose significant body mass and become nutritionally depleted with hypoalbuminaemia and refractory anaemia [7]. Advanced cases can also present acutely, with abdominal collections, haemoperitoneum, or bowel perforation leading to peritonitis and sepsis. However, the presentation can also be non-specific with intermittent symptoms, and combined with the fact that most clinicians may never encounter EPS, diagnosis may be difficult or delayed [8]. Risk factors for the development of EPS secondary to PD include starting PD at a younger age, duration of PD, and episodes of peritonitis [9]. There is currently no consensus or guideline on the management of EPS. Suggested treatment includes cessation of PD, medical therapy in the form of immunosuppressive or antifibrotic agents and surgical intervention. Controversy exists among the limited literature regarding the efficacy, safety and outcomes related to these treatments [10]. Enteral or parenteral nutrition (PN) can be important to prevent malnutrition and has been shown to improve outcomes following surgery for EPS [11]. Reported mortality of EPS remains high, ranging between 25 and 56% in adult studies [12].

EPS in the paediatric population has been described in individual case reports or reviews of registries to establish incidence and mortality. There remains a scarcity in the literature on surgical care and outcomes. The Manchester Centre For Transplantation (UK) has previously published a case report of a 16-year-old boy treated successfully with EPS surgery [13]. As an international referral centre, this novel report summarises our experience of all paediatric patients undergoing surgical intervention. We highlight clinical challenges surrounding diagnosis, clinical management, and outcomes and reflect on the current literature to provide meaningful recommendations.

## Case series

We reviewed the Manchester EPS database and identified four patients below the age of 16 years at the time of surgery. The series included a single patient from the UK, with three further patients from the Netherlands, Bosnia and Herzegovina, and Greece. Demographics, patient characteristics, and medical history are displayed in Table 1 and the clinical course, surgical management and outcomes are summarised in Table 2. All clinical data was managed in accordance with the standards of research set by the Declaration of Helsinki. All patients provided explicit, written consent.

**Table 1:** Summary of patient characteristics and medical history of paediatric patients with EPS managed at Manchester

Number of patients (n)	4
Male	3/4 (75%)
Primary disease (n)	FSGS (1), bilateral Wilms’ tumours (1), neurogenic bladder (1), posterior urethral valves (1)
Age PD commenced (months–median (range))	44.5 (1–108)
Duration of PD (months–median (range))	78 (60–108)
Age at diagnosis of EPS (months–median (range))	126 (84–192)
Recurrent peritonitis^1^	4/4 (100%)
EPS following cessation of PD	4/4 (100%)
Diagnostic criteria (n)	CT-scan (4)
Urgency (n)	Emergency (2), semi-elective (2)
Dialysis modality at the time of EPS surgery	Transplant (1), haemodialysis (3)
Duration of PN (days–median (range))	30 (0–46) days
Post-operative morbidity	2/4 (50%)
Stoma formation	2/4 (50%)
Primary closure of the abdomen	2/4 (50%)
Mortality	0/4 (0%)
Length of stay (days–median (range))	25 (19–63)
Recurrence	0/4 (0%)
Current dialysis modality (n)	Transplant (2), haemodialysis (2)

^1^Recurrent PD defined as > 3 episodes

**Table 2:** Case summaries including clinical course, surgical management and outcomes

*Case no*	Clinical presentation	Medical treatment	Surgical management	Outcome	Follow-up
1	Bowel obstruction	None	**Findings**: generalised EPS, fibrotic cocoon, ascites **Procedure**: Laparotomy + lavage + enterolysis	No recurrence of EPS, normal growth and development, functioning transplant	12 years
2	Bowel obstruction	Steroids and colchicine	**Findings**: generalised EPS, bowel perforation, ascites **Procedure**: laparotomy + lavage + enterolysis + catheter ileostomy + 4 quadrant drain insertion	No recurrence of EPS, normal growth and development, functioning transplant	10 years
3	Non-specific	None	**Findings**: generalised EPS, fibrotic cocoon, bowel perforation **Procedure**: laparotomy + lavage + enterolysis + double barrelled ileostomy – abdomen left open	No recurrence of EPS, normal growth and development, awaiting transplant	9 years
4	Bowel obstruction	Tamoxifen (changed to cyclosporine 3 years later) and steroids	**Findings**: generalised EPS, fibrotic cocoon, ascites **Procedure**: Laparotomy + lavage + enterolysis – abdomen left open	No recurrence of EPS, normal growth and development, awaiting transplant	2 years

### Case 1

A 13-year-old boy with a background of focal segmental glomerulosclerosis (FSGS), hypertension, previous failed kidney transplant (recurrent disease) presented to their local hospital with acute bowel obstruction two months after the change of PD to haemodialysis for progressive ultrafiltration failure (UF). Initially, he was managed with intravenous fluids and nil orally. However, he developed tense ascites with worsening abdominal pain and bilious vomiting and was thus transferred to Manchester for further management. He underwent emergency laparotomy due to worsening sepsis, where generalised EPS was confirmed with his entire small bowel encased in a fibrotic cocoon and large volume ascites. A complete enterolysis was performed, and the abdomen was thoroughly lavaged. Post-operatively, oral intake was gradually increased over two weeks until parental nutrition (PN) independence was established. The patient subsequently underwent a live donor transplant five years later and is now 12 years since EPS surgery. He remains well with no recurrence of EPS or abdominal symptoms and a functioning graft.

### Case 2

**Fig. 1:**
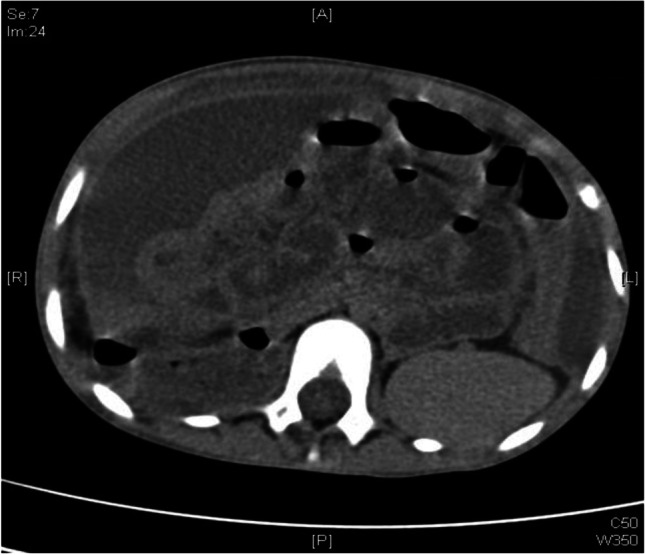
Pre-operative CT-scan of *Case 2* showing profuse turbid ascites, thickening of the intestinal wall, intestine tethered to the spinal column, but no peritoneal calcifications

A seven-year-old girl with kidney failure secondary to bilateral nephrectomy due to bilateral Wilms’ tumours presented with bowel obstruction three months after a living donor kidney transplant. A diagnosis of EPS was made on CT-scan (Fig. 1). The child had undergone a diagnostic laparoscopy at the referring hospital. The bowel was found to be covered in fibrotic connective tissue with acute on chronic inflammatory changes and ascites. EPS was managed medically over 12 months, during which there were multiple acute episodes managed with high-dose steroid therapy. Fifteen months after transplantation, the patient was admitted as an emergency to the referring centre with peritonitis and diagnosed with bowel perforation. She was transferred to Manchester by air ambulance for further management and underwent surgery immediately due to worsening sepsis.

At surgery, a single bowel perforation and free enteric content was confirmed. A complete enterolysis was carried out and the abdomen thoroughly lavaged. As the perforation was small, a decision was made to carry out a catheter ileostomy rather than a standard ileostomy. The abdomen was closed with four-quadrant wide-bore surgical drains.

After initial improvement, the catheter stoma became detached from the abdominal wall leading to a peritoneal collection associated with rising inflammatory markers. A second laparotomy was undertaken 13 days after the initial procedure through the same incision. On the same occasion, a standard loop ileostomy was carried out. Subsequently, the patient gradually increased oral intake and was discharged at 23 days. The ileostomy was reversed after three months, and the patient remains well at 10 years of follow-up, with no recurrence of EPS and a functioning transplant.

### Case 3

A 16-year-old boy with a background of neurogenic bladder and reflux nephropathy presented with abdominal distension, vomiting, and weight loss three months following a change of PD to haemodialysis for UF failure. After a short course of conservative management, a laparotomy was performed via a midline incision at the referring centre. The small bowel was found to be covered in a thick cocoon that could not be separated from the intestinal wall. During the procedure, there was an iatrogenic perforation of the mid-ileum that was managed with seromuscular, single-layered sutures. During the post-operative course, an enteric fistula developed, and PN was commenced. The patient was then transferred to Manchester for specialist management.

After a two-week period of stabilisation with PN, regular dialysis, and antibiotics, the abdomen was re-explored. There was a cavity anterior to the cocoon extending into the left hypochondrium containing feculent material. A punched-out perforation was seen in the cocooned mass. After complete enterolysis, the perforation was excised and was brought out in the left iliac fossa as a double-barreled ileostomy. The patient was kept ventilated in the pediatric ICU and, after two days, reexplored. The gut was found to be healthy without any evident perforations. The abdomen was closed with a non-crosslinked porcine dermal matrix biologic mesh (STRATTICE™, AbbVie©, Illinois, USA) with skin closure over the mesh. A vacuum dressing was placed over the closure. There was a six-week recuperation period, complicated by wound sepsis with both Vancomycin-Resistant Enterococcus (VRE) and *Candida*, the latter requiring micafungin for three weeks. A full oral diet was established after 10 days, and PN was discontinued.

After 6 months, the patient returned to Manchester for planned elective closure of the stoma. A midline laparotomy was carried out, and the abdomen was inspected. The gut was found to be healthy without any evidence of cocooning or adhesions. After initially doing well, the patient developed an enterocutaneous fistula toward the bottom of the laparotomy incision after one week. This was treated conservatively with PN for eight weeks, at the end of which the fistula healed and full oral diet established. At nine years, the patient remains well on hemodialysis awaiting a transplant.

### Case 4

**Fig. 2:**
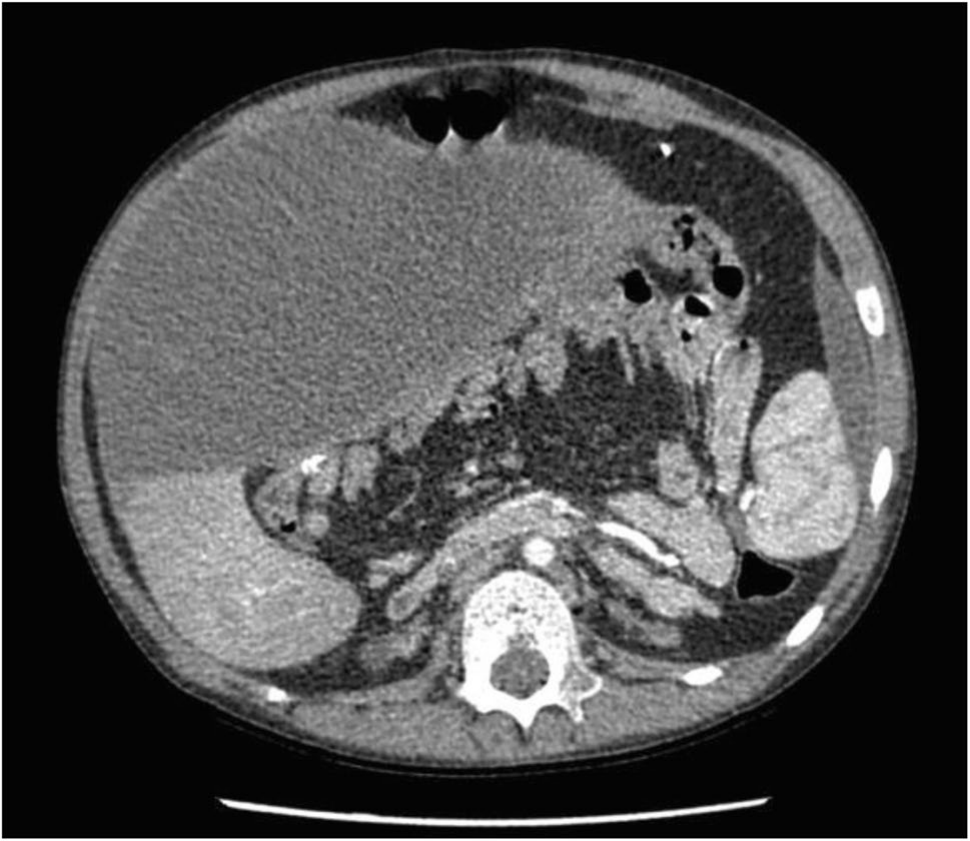
Pre-operative CT scan of *Case 4* showing a large encapsulated abdominal fluid collection with associated peripheral peritoneal calcification

An eight-year-old boy with kidney failure secondary to posterior urethral valves and reflux nephropathy presented with severe abdominal pain and abdominal distention, accompanied by vomiting, constipation and fever one month after the discontinuation of PD, due to progressive UF failure. Sub-acute bowel obstruction secondary to EPS was confirmed on abdominal CT scan. This demonstrated the presence of loculated ascites, accompanied by adhesion of bowel loops and a few areas of peritoneal calcification (Fig. 2). The patient was admitted to the local ICU and treated with PN, frequent albumin infusions, opioid analgesics and intravenous antibiotics, including aminoglycoside, metronidazole and a third-generation cephalosporin. EPS treatment with steroids and tamoxifen was introduced, and the patient recovered fully for one month. After the first episode, the patient continued with medical treatment and was followed up with a CT scan after 12 months. This revealed stable findings of a large abdominal encapsulated fluid collection. Of note, tamoxifen was changed to cyclosporine after three years due to the development of aplastic anemia that was not responsive to erythropoietin. During the four-year follow-up period, the patient presented with four further episodes of acute abdominal pain and vomiting, attributed to sub-acute bowel obstruction. These were successfully managed with intravenous antibiotics, nil by mouth, analgesics, and intermittent increase in steroid dose. However, due to the regularity of acute exacerbations, the patient was referred to Manchester for definitive surgical management.

**Fig. 3:**
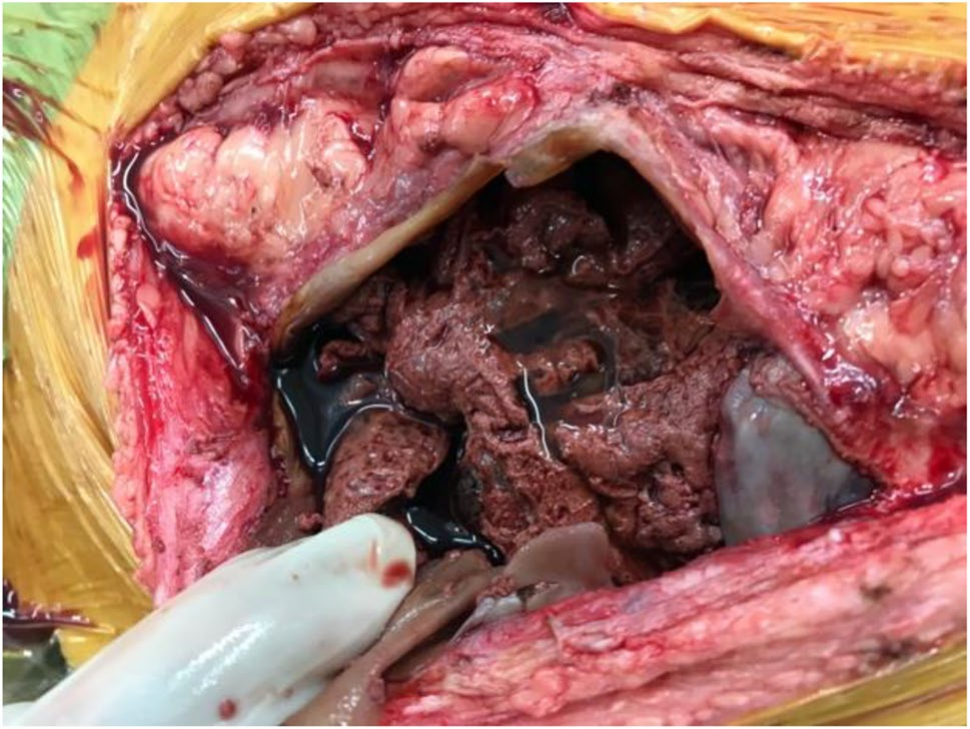
Intra-operative picture of index procedure at the first inspection of the abdomen (*Case 4*), demonstrating bloodstained ascites and fibrous encasement of the small bowel

**Fig. 4:**
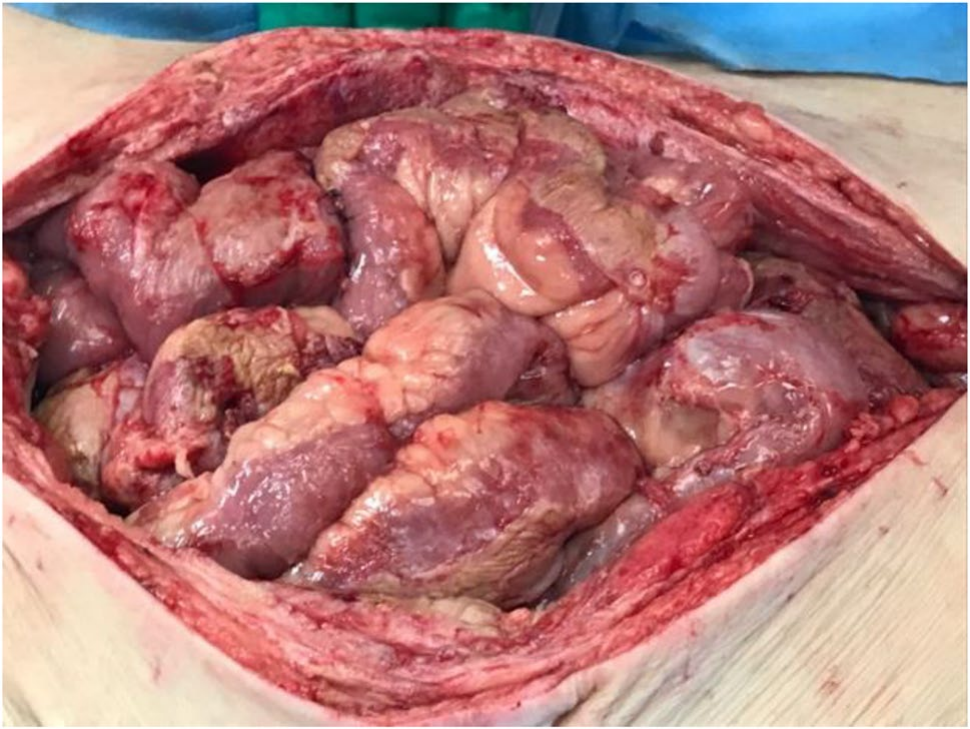
Intra-operative picture at 48 h post-index procedure (C*ase 4*), showing viable small bowel loops and healthy abdominal wall tissue for safe closure

At Manchester, the patient underwent a laparotomy and was found to have generalised EPS with small bowel encased in a fibrotic cocoon and large volume ascites (Fig. 3). No faecal contamination or perforation was encountered, and complete enterolysis performed. However, due to concerns over the tension of the closure, the abdomen was left open for two days as a laparostomy. At re-look, the abdomen was found to be clean with healthy bowel and no residual collection; thus, the abdomen was closed (Fig. 4*)*. Oral intake was established promptly. PN was not required throughout the admission. The patient remains well with no recurrence of EPS and is currently on haemodialysis awaiting a transplant.

## Discussion

This is the first paediatric case series on definitive surgical intervention for EPS. It highlights the complexity and variability in clinical course, operative findings, and surgical management. Despite the high incidence of post-operative morbidity, surgery offered definitive treatment, with no long-term recurrence and 100% survival. Previous registry-based studies from Japan (11 cases), Italy (14 cases) and Europe (EU-wide) (22 cases) have reviewed EPS in children and are discussed below, including challenges around diagnosis and referral, medical and surgical treatment and long-term outcomes [14–16].

There is currently no gold standard for the diagnosis of EPS. However, a high index of suspicion should be maintained in patients on long-term PD (> 5 years) who present with abdominal symptoms, malnutrition and raised inflammatory markers [17]. Recurrent episodes of peritonitis, especially if culture results indicate *Staphylococcus aureus*, *Enterococcus*, *Pseudomonas* or fungi, have been shown to increase the risk of EPS in children [3]. The overall prevalence of EPS paediatric patients is reported to be low at 1.5% to 1.9% across the three registry studies [14–16]. These findings are similar to data from adult PD cohorts [2, 3]. All three registry studies found an association of EPS with increasing time on PD in children; however, the Japanese study did not find an association with recurrent episodes of peritonitis [14–16]. In our series, all patients received PD for over five years and had recurrent episodes of peritonitis. However, EPS remains a rare phenomenon and Goodlad et al. investigated the role of CT screening in long-term PD treatment identifying no association between CT scan findings and clinical EPS in asymptomatic patients [18].

Patients in our series typically presented following cessation of PD with abdominal symptoms related to bowel obstruction. In three cases, PD was switched to HD due to progressive failure of UF. Previous studies have postulated that it may be early EPS that leads to UF failure — rather than discontinuation of PD being the cause of EPS [19]. Similar to adult studies, the non-specific nature of symptoms means that children with EPS can suffer with abdominal symptoms for long periods before the diagnosis is made [8, 17]. Early referral for specialist assessment has therefore been recommended by the EPS registry guidelines from The Netherlands [20]. However, the EU-wide and Italian registry studies found that over 70% of children suffered from malnutrition, suggesting advanced disease with a significant impact of EPS symptoms on oral intake [15, 16]. In our series, three out of four patients were referred to Manchester within six months of diagnosis. However, two of the patients presented with acute abdominal symptoms due to bowel perforation and required emergency surgical intervention. Adult reports suggest that EPS surgery in the emergency setting significantly increases morbidity leading to poor outcomes and mortality. Therefore, maintaining a high index of suspicion, but also seeking specialist input may allow early assessment and planned surgical intervention [21].

Medical therapies have been used to treat EPS in both adults and children [17, 22]. Steroid therapy is the most commonly-reported medical therapy in the paediatric registry studies (Japan — 2/11, Italy — 6/14 and EU-wide — 17/22) [14–16]. Steroids were administered in two patients in our series and EPS managed medically for one and four years respectively, prior to the need for surgical intervention. Tamoxifen has also been reported to successfully treat or reduce the morbidity associated with EPS in adult case series and was used in one patient in our series [23, 24]. However, no patient in the Japanese study and only 1/9 and 9/22 children in the Italian and EU-wide studies received Tamoxifen [14–16]. As such, no consensus guidelines on medical treatment of EPS exist as yet. In our experience, trial of medical therapy in non-acute presentations should be combined with early referral to specialist centres. This will ensure close contact with surgical expertise in case the clinical condition of the patient worsens or an emergency arises.

Surgical intervention offers definitive treatment. In our series, surgery was performed either for acute presentations with bowel perforation or obstruction (Cases 1, 2 and 3) or due to recurrent exacerbation of subacute obstruction requiring hospital admission and intensive medical therapy (Case 4). Based on our experience and previously published adult literature, a multi-disciplinary approach and pre-operative optimisation with parenteral nutrition, where possible, is recommended [25]. De Freitas et al. highlighted the importance of nutritional management in adult patients undergoing surgery for EPS, and we propose that these principles be translated to paediatric patients [11].

Outcomes of paediatric EPS surgery have only been reported in the Italian registry study with a mortality of 50% (3/6). All three deaths were related to recurrent bowel perforation following surgical intervention [15]. A single patient in the Japanese series and 14 patients in the EU-wide series were treated with surgery, but their outcomes have not been reported [14, 16]. Overall mortality from EPS across all registry studies was still reported to be high (though lower than in adult studies): Japan — 27%, Italy — 43% and EU-wide — 13.6%) [14–16].

Surgery for EPS is associated with significant perioperative morbidity. Fibrosis of the peritoneum leads to bowel loops being fused, with no anatomical plane to dissect. To release the bowel and relieve obstruction, enterolysis (dissection of adhesions) must be performed. Best outcomes have been shown if the entire length of small bowel from duodenal flexure to ileocaecal valve is dissected free. This is a time-consuming task with high risk of iatrogenic perforation. A Japanese series of 50 adult patients undergoing EPS surgery found a mean operating time of just under 7 h [25]. A more recent review from the same group reflecting on their experience recommended that EPS surgery should be performed in specialist high-volume centres [26].

In our experience, meticulous sharp dissection with a scalpel and scissors proves the safest technique with the least risk of perforation. The risk of post-operative complications remains high, with two patients in our series developing post-operative enteric fistulae at some point. Our specific surgical experience and expertise allowed improved intra-operative decision-making regarding stoma formation and primary closure versus leaving the abdomen open as a laparostomy with a planned closure at 24 to 72 h. The use of laparostomy and re-look has been well-reported in adult patients and we would recommend this method to reduce the risk of intra-abdominal compartment syndrome when deemed necessary [27]. Re-establishing oral intake is the primary goal of surgery and all patients in our series were supported with PN until full oral intake was established before discharge.

Follow-up in our series ranged from 2 to 12 years. All patients are alive, with two patients having functioning grafts. We propose that EPS, which is a rare complication of prolonged PD exposure, appears to be safely treated with surgical intervention in children. Surgery should be undertaken in a planned semi-elective manner, as emergency intervention may lead to poorer outcomes. Meticulous technique and supporting infrastructure appear to be critical in successful outcomes. It is likely that best results are achieved in centres experienced with the management of this challenging condition. Subsequent transplantation is feasible, safe and the ideal kidney replacement therapy with EPS surgery providing the bridge to successful transplantation.

With this report, we hope to contribute to the literature on this rare condition in children, alerting paediatric physicians caring for PD patients to consider the diagnosis of EPS and involve specialist input at the earliest possible time. Well-timed surgical enterolysis in our experience is perhaps the only definitive and effective treatment option for this condition.

## Supplementary Information

Supplementary file1 (pptx 76.5 KB)

## Data Availability

All data in this study has been de-identified and anonymised. All data pertaining to this study is made available in the body of the manuscript.
