# Supplementary material for: Surgical management of Encapsulating Peritoneal Sclerosis (EPS) in children: international case series and literature review
Source: Pediatr Nephrol. 2021 Aug 26;37(3):643–50. doi: 10.1007/s00467-021-05243-0 (PMC8921033; doi:10.1007/s00467-021-05243-0)
Supplement: Supplementary file 1 — Supplementary file1 (pptx 76.5 KB) [file 467_2021_5243_MOESM1_ESM.pptx]

## Slide 1
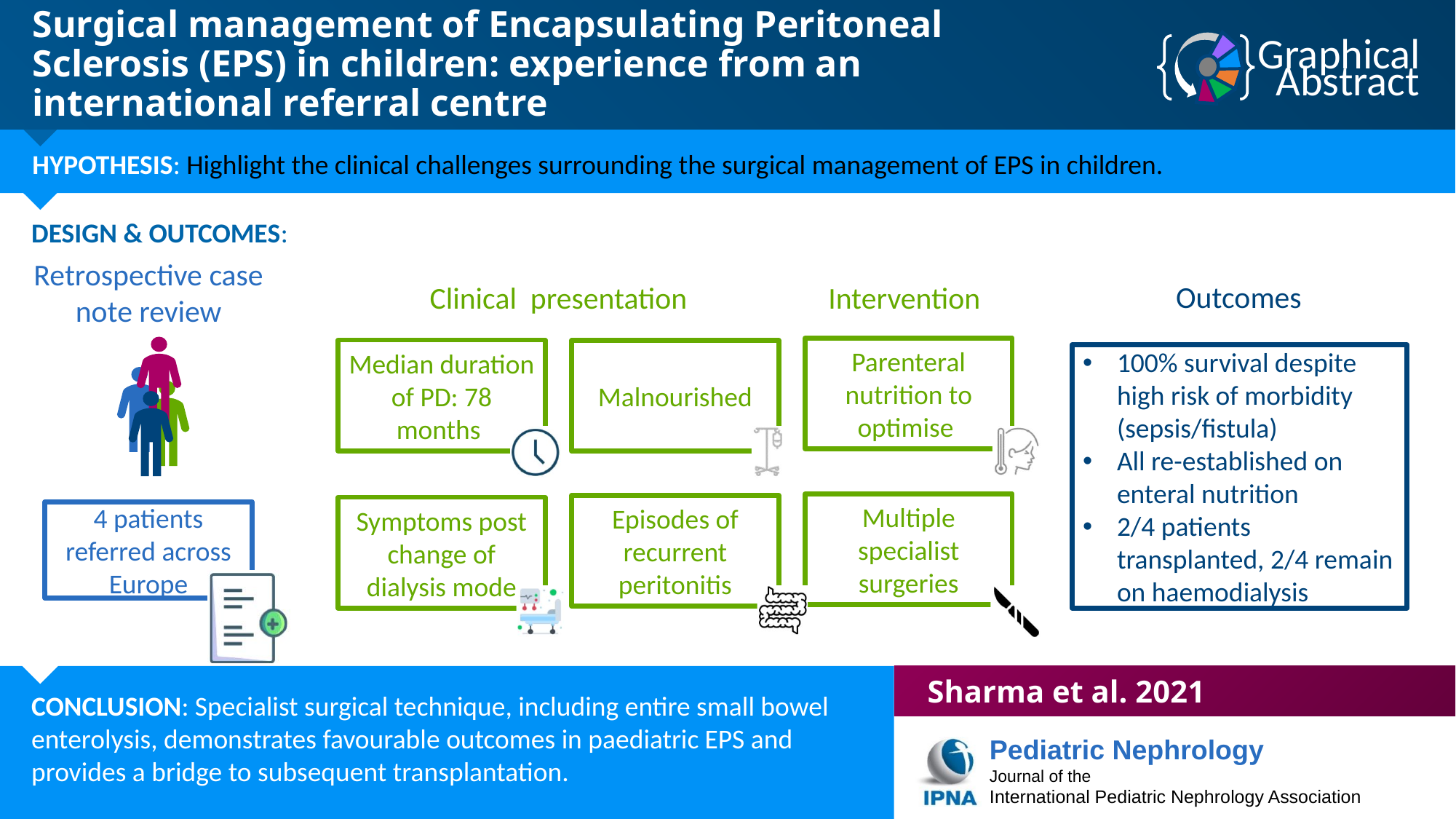

Surgical management of Encapsulating Peritoneal
Sclerosis (EPS) in children: experience from an
international referral centre
HYPOTHESIS: Highlight the clinical challenges surrounding the surgical management of EPS in children.
DESIGN & OUTCOMES:
Retrospective case note review
Outcomes
Clinical presentation
Intervention
Parenteral nutrition to optimise
Median duration of PD: 78 months
Malnourished
100% survival despite high risk of morbidity (sepsis/fistula)
All re-established on enteral nutrition
2/4 patients transplanted, 2/4 remain on haemodialysis
Multiple specialist surgeries
Episodes of recurrent peritonitis
Symptoms post change of dialysis mode
4 patients referred across Europe
Sharma et al. 2021
CONCLUSION: Specialist surgical technique, including entire small bowel enterolysis, demonstrates favourable outcomes in paediatric EPS and provides a bridge to subsequent transplantation.
